# Adrenocorticotropin-secreting pituitary macroadenomas: expanding the clinical spectrum

**DOI:** 10.1210/jcemcr/luag127

**Published:** 2026-05-25

**Authors:** Caitlin Iyer, Nicola Havenga, David Roytowski, Razaan Davis, Dan Zaharie, Marli Conradie-Smit

**Affiliations:** Division of Endocrinology, Department of Medicine, Faculty of Medicine and Health Sciences, Tygerberg Hospital and Stellenbosch University, Cape Town 7505, South Africa; Division of Endocrinology, Department of Medicine, Faculty of Medicine and Health Sciences, Tygerberg Hospital and Stellenbosch University, Cape Town 7505, South Africa; Department of Neurosurgery, Faculty of Medicine and Health Sciences, Tygerberg Hospital and Stellenbosch University, Cape Town 7505, South Africa; Division of Radiodiagnosis, Department of Medical Imaging and Clinical Oncology, Faculty of Medicine and Health Sciences, Stellenbosch University, Cape Town 7505, South Africa; Division of Anatomical Pathology, Department of Pathology, National Health Laboratory Services, Tygerberg Hospital and Stellenbosch University, Cape Town 7505, South Africa; Division of Endocrinology, Department of Medicine, Faculty of Medicine and Health Sciences, Tygerberg Hospital and Stellenbosch University, Cape Town 7505, South Africa

**Keywords:** Cushing disease, ACTH-secreting pituitary macroadenoma, hypopituitarism, transsphenoidal surgery

## Abstract

Cushing disease (CD) secondary to adrenocorticotropin (ACTH)-secreting pituitary macroadenomas is uncommon, accounting for 10% to 15% of cases. We describe 3 patients from South Africa who presented with overt clinical features of hypercortisolism and considerable systemic complications, including hypertension, diabetes mellitus, and osteoporosis. Biochemical testing confirmed ACTH-dependent hypercortisolism with hypopituitarism and mild hyperprolactinemia consistent with stalk effect. Magnetic resonance imaging demonstrated pituitary macroadenomas, measuring 10 mm or greater, with parasellar extension. Mass effect was observed in 1 patient resulting in cranial nerve III palsy, while the others had visual field deficits secondary to optic chiasm compression. All patients underwent endoscopic transsphenoidal surgery, with 1 achieving remission and 2 developing recurrence. These cases highlight that ACTH-secreting macroadenomas can present with severe systemic disease and significant mass effect, and emphasize the importance of lifelong follow-up.

## Introduction

Cushing syndrome (CS) refers to a clinical state of chronic cortisol excess with Cushing disease (CD) being the most common endogenous cause. CD is caused by excessive adrenocorticotropin (ACTH) secretion by a monoclonal pituitary corticotroph adenoma, now referred to as a pituitary neuroendocrine tumor, and has an incidence of 1.2 to 2.4 new cases per million per year [1]. Pituitary microadenomas (<10 mm) [2] account for approximately 80% to 90% of cases [2], whereas macroadenomas (≥10 mm) [3] are uncommon, accounting for only 10% to 15% of cases [3]. Microadenomas are usually confined to the sella turcica [2], while macroadenomas are more invasive and carry a higher risk of cavernous sinus or parasellar extension and associated mass effect [3, 4].

There is limited published literature on ACTH-secreting macroadenomas, particularly focusing on comparing macroadenomas and microadenomas [4-9]. These studies highlight uncertainty regarding diagnostic reproducibility of the biochemical results and the correlation between tumor size, serum cortisol levels, and serum ACTH levels [4-9]. Transsphenoidal surgery (TSS) remains first-line treatment for functional macroadenomas [2].

We present 3 cases of ACTH-secreting pituitary macroadenomas from South Africa. To our knowledge, this is the first case series reported from Sub-Saharan Africa. These cases highlight severe systemic complications, mass effect, perioperative complications, and recurrence. This series further emphasizes that ACTH-secreting macroadenomas may present with severe clinical disease, in contrast to prior studies reporting milder clinical manifestations [4].

## Case presentation

### Case 1

A 60-year-old woman with hypertension and osteoporosis presented with abdominal pain, subsequently diagnosed as diverticulitis, with a history of sudden weight gain, easy bruising, and visual disturbances.

### Case 2

A 32-year-old woman with hypertension, type 2 diabetes mellitus, and low bone density for age (with multiple fragility fractures) presented with congestive cardiac failure (CCF). She had a history of easy bruising, acne, weight gain, muscle weakness, and a depressed mood.

### Case 3

A 56-year-old woman with hypertension, type 2 diabetes mellitus, and osteopenia presented with a 2-year history of headaches, blurry vision, dizziness, and syncopal episodes, followed by sudden weight gain, shortness of breath, bruising, and muscle weakness. Past medical history included a total thyroidectomy for a multinodular goiter (Bethesda IV) with compressive symptoms, for which she was on levothyroxine.

## Diagnostic assessment

### Case 1

**Figure 1:**
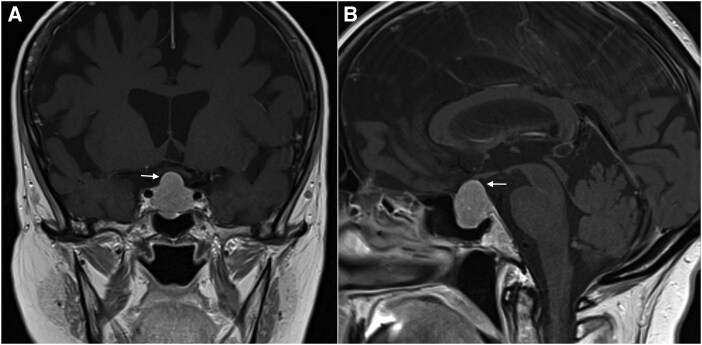
A, T1-weighted (T1W) postcontrast coronal magnetic resonance imaging (MRI) demonstrates a homogeneously enhancing mass arising from and expanding the sella turcica. It extends into the suprasellar cistern and abuts the optic chiasm (arrow). The mass extends to the right internal carotid artery, between the medial tangent and intercarotid lines (Knosp grade 1). B, T1W postcontrast sagittal MRI demonstrates the expanded sella turcica and the convex superior border (arrow).

Clinically, the patient had central obesity, facial plethora, bruising, proximal myopathy, and left-sided visual field loss. Biochemical testing confirmed CD revealing loss of circadian rhythm in the hypothalamic-pituitary-adrenal axis, ACTH-dependent hypercortisolism, hypogonadotropic hypogonadism, secondary hypothyroidism, and mild hyperprolactinemia (Table 1). Dynamic testing using high-dose dexamethasone suppression testing (DST) was deferred due to overt cortisol excess (serum nocturnal cortisol and urinary free cortisol [UFC]), which was diagnostic [10]. Osteoporosis was confirmed on dual-energy x-ray absorptiometry (DXA) with a T-score of −2.6 for L3 and L4 vertebral bodies. Biconcave deformity fractures were found in L2 and L3 vertebral bodies. Magnetic resonance imaging (MRI) demonstrated a pituitary macroadenoma, measuring 17 × 19 × 22 mm, extending into the suprasellar region (Fig. 1A and 1B) and compressing the optic chiasm, clinically manifesting as visual field loss [11]. Generalized cerebral atrophy, out of keeping with age, was also noted and likely due to accelerated cerebral aging linked to hypercortisolism [2].

**Table 1:** Laboratory results for the patient cases

Laboratory value	Case 1	Case 2	Case 3
08:00 Serum cortisol RR: 4.8-19.5 µg/dL (SI: 133-537 nmol/L)	**31.7 µg/dL** (SI: 875 nmol/L)	**49.7 µg/dL** (SI: 1372 nmol/L)	**28.0 µg/dL** (SI: 772 nmol/L)
23:00 Serum cortisol RR: 2.5-11.9 µg/dL (SI: 68-327 nmol/L)	**27.7 µg/dL** (SI: 764 nmol/L)	**52.1 µg/dL** (SI: 1437 nmol/L)	**19.4 µg/dL** (SI: 535 nmol/L)
UFC (24 h) RR: 3.6-45 µg/24 h (SI: 10-124 nmol/24 h)	**638 µg/24 h** (SI: 1760nmol/24 h)	**1581 µg/24 h** (SI: 4358 nmol/24 h)	**102 µg/24 h** (SI: 280 nmol/24 h)
1-mg DST cortisol RR: <1.8 µg/dL (SI: <50 nmol/L)	Not done	Not done	**14.9 µg/dL** (SI: 411 nmol/L)
ACTH RR: 7.3-63.9 pg/mL (SI: 1.6-13.9 pmol/L)	57.2 pg/mL (SI: 12.6 pmol/L)	**197 pg/mL** (SI: 43.4 pmol/L)	**102.5 pg/mL** (SI: 22.6 pmol/L)
FSH RR: 25.8-134.8 IU/L	**0.5 IU/L**	**0.2 IU/L**	**1.4 IU/L**
LH RR: 7.7-58.5 IU/L	**<0.1 IU/L**	**<0.1 IU/L**	**<0.1 IU/L**
TSH RR: 0.27-4.20 mIU/L	**0.01 mIU/L**	0.99 mIU/L	2.09 mIU/L (on levothyroxine)
FT4 RR: 0.9-1.7 ng/dL (SI: 12.0-22.0 pmol/L)	0.96 ng/dL (SI: 12.4 pmol/L)	**0.61 ng/dL** (SI: 7.9 pmol/L)	1.44 ng/dL (SI: 18.5 pmol/L) (on levothyroxine)
Prolactin RR: 4.8-23.3 ng/mL (SI: 4.8-23.3 µg/L)	**42.7 ng/mL** (SI: 42.7 µg/L)	**27.1 ng/mL** (SI: 27.1 µg/L)	**111 ng/mL** (SI: 111 µg/L)
HbA_1c_	5.4%	**6.8%**	**12.1%**
RR: <6.5%			
Serum creatinine RR: 0.55-1.02 mg/dL (SI: 49-90 µmol/L)	0.88 mg/dL (SI: 78 µmol/L)	0.66 mg/dL (SI: 58 µmol/L)	0.89 mg/dL (SI: 79 µmol/L)
Serum calcium RR: 8.8-10.2 mg/dL (SI: 2.2-2.55 mmol/L)	**8.28 mg/dL** (SI: 2.07 mmol/L)	9.6 mg/dL (SI: 2.4 mmol/L)	**8.72 mg/dL** (SI: 2.18 mmol/L)

Values in bold indicate results outside the reference range.

Abbreviations: ACTH, adrenocorticotropin; DST, dexamethasone suppression test; FSH, follicle-stimulating hormone; FT4, free thyroxine; HbA_1c_, glycated hemoglobin A_1c_; LH, luteinizing hormone; RR, reference range; TSH, thyrotropin; UFC, urinary free cortisol.

### Case 2

**Figure 2:**
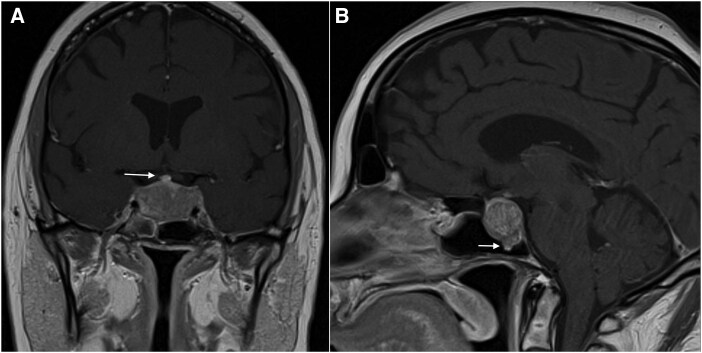
A, T1-weighted (T1W) postcontrast coronal magnetic resonance imaging (MRI) demonstrates a heterogeneously enhancing mass and an enhancing undisplaced pituitary stalk (arrow). The mass extends into the suprasellar cistern with minimal compression of the optic chiasm. Laterally it partially encases the right and left internal carotid arteries, extending lateral to the lateral tangent lines, in keeping with bilateral Knosp grade 3. B, T1W postcontrast sagittal MRI demonstrates the expanded sella turcica with a small nubbin of tissue (arrow) extending into the right sphenoid sinus.

The patient presented with CCF and signs of excessive cortisol including bruising, thinning of the skin, widespread violaceous striae, facial plethora, hirsutism, acne, central obesity, and proximal muscle weakness. She also reported a depressed mood, in keeping with psychiatric manifestations of CD [1]. Biochemistry confirmed ACTH-dependent hypercortisolism, hypogonadotropic hypogonadism, secondary hypothyroidism, and mild hyperprolactinemia (see Table 1). DXA revealed the patient's bone mass was abnormally low for age, sex, and ethnicity, with multiple thoracic and lumbar vertebral body fractures and a Z-score of −3.4 for L1 to L4 vertebral bodies. MRI demonstrated a pituitary macroadenoma, measuring 17 × 25 × 21 mm. Minimal compression of the optic chiasm, right sphenoid sinus invasion, and partial encasement of the internal carotid arteries (ICAs) in the cavernous sinuses bilaterally (Fig. 2A and 2B) was seen (Knosp grade 3). Neurological examination revealed a left-sided ptosis with a dilated pupil, consistent with cranial nerve III palsy [4, 11].

### Case 3

**Figure 3:**
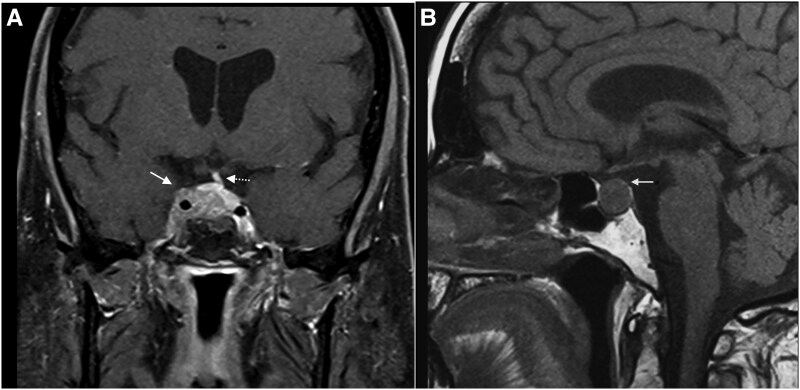
A, T1-weighted (T1W) postcontrast coronal magnetic resonance imaging (MRI) demonstrates a heterogeneous mass located on the right (solid arrow), infiltrating the cavernous sinus and encasing the right internal carotid artery (Knosp grade 4). The mass enhances to a lesser degree than the normal pituitary gland and the pituitary stalk is displaced to the left (dashed arrow). B, T1W postcontrast sagittal MRI demonstrates the expanded sella turcica and the mass, which extends into the suprasellar cistern (solid arrow), but does not compress or displace the optic chiasm.

The patient, with class III obesity, had facial plethora, moon facies, a dorsocervical fat pad, multiple bruises, violaceous striae on the abdomen, and proximal lower limb myopathy. No neurological deficits were noted. Biochemistry confirmed ACTH-dependent hypercortisolism with hypogonadotropic hypogonadism and mild hyperprolactinemia, consistent with stalk effect (see Table 1) [11]. Due to her prior thyroidectomy, the intrinsic thyroid axis could not be assessed, as hormone levels are determined by exogenous replacement. Thyroid function was adequate on levothyroxine replacement. Osteopenia of the left hip (T-score −1.1) was found on DXA. MRI of the sella demonstrated a pituitary macroadenoma, measuring 12 × 19 × 10.2 mm, located on the right, infiltrating the cavernous sinus (Fig. 3A and 3B) and encasing the right ICA (Knosp grade 4). The pituitary stalk was displaced to the left.

## Treatment

### Case 1

The patient was started on levothyroxine 50 mcg daily for secondary hypothyroidism and received 4 mg of zoledronic acid intravenously for osteoporosis. Preoperatively, she was initiated on cabergoline 250 mcg twice weekly and ketoconazole 200 mg every 12 hours.

She underwent successful endoscopic TSS, with partial resection of the mass due to significant extension. Perioperative management included intravenous hydrocortisone 50 mg every 8 hours, which was weaned in the hospital prior to discharge.

### Case 2

The patient underwent endoscopic TSS with resection of the entire tumor. Postoperatively, the left-sided ptosis and cranial nerve palsy resolved. The patient received 4 mg of zoledronic acid intravenously for osteoporosis. Hydrocortisone was not given perioperatively, and the patient developed acute cortisol withdrawal syndrome in the form of palpitations, severe tremors, hallucinations, and severe depression, despite persistently high serum cortisol values. The patient was started on intravenous hydrocortisone 50 mg every 6 hours, which was weaned to oral hydrocortisone 10 mg daily and continued.

### Case 3

The patient underwent uncomplicated endoscopic TSS. Postoperatively, she had no visual deficits. She was initiated on intravenous hydrocortisone 50 mg every 8 hours perioperatively, which was weaned prior to discharge. For her osteoporosis, she received a single 4-mg dose of intravenous zoledronic acid.

## Outcome and follow-up

### Case 1

Histopathology confirmed an ACTH-secreting pituitary tumor. One month postoperatively, the patient had clinical and biochemical improvement, with a low morning serum cortisol (4.9 µg/dL [SI: 136 nmol/L]) (reference range [RR] 4.8-19.5 µg/dL [SI: 133-537 nmol/L]), and suppression on DST (<1.8 µg/dL [SI: <50 nmol/L]) (RR <1.8 µg/dL [SI: <50 nmol/L]). UFC normalized (22.5 µg/24 hours [SI: 62 nmol/24 hours]) (RR 3.6-45 µg/24 hours [SI: 10-124 nmol/24 hours]) on 6-month follow-up. Her blood pressure and weight improved, and MRI demonstrated a smaller pituitary adenoma with resolution of optic chiasm compression.

After 4 years, she developed recurrent CD due to regrowth of the residual tumor. She underwent repeat TSS followed by remission.

### Case 2

Histopathology confirmed an ACTH-producing pituitary tumor. Biochemical markers normalized, with a morning serum cortisol of 17.6 µg/dL (SI: 485 nmol/L), suppression on DST (1.6 µg/dL [SI: 44 nmol/L]), and on follow-up several months postoperatively, a normal UFC of 34.1 µg/24 hours (SI: 94 nmol/24 hours). Clinical features resolved, and MRI revealed no residual disease. Her menstrual cycle returned and there was adequate glycemic control off treatment, although the hypertension persisted. Hydrocortisone replacement (10 mg morning and 5 mg afternoon) was tapered, and levothyroxine 25 mcg daily was continued.

### Case 3

One month postoperatively, the patient’s morning serum cortisol normalized (17.4 µg/dL [SI: 480 nmol/L]). Thyroid function, glycemic control, and blood pressure control were adequate on levothyroxine, metformin, a basal bolus insulin regimen, and antihypertensives. Six months postoperatively, the patient developed disease recurrence with an elevated UFC (158.7 µg/24 hours [SI: 438 nmol/24 hours]) and morning serum cortisol (22.9 µg/dL [SI: 631 nmol/L]). Further management is pending.

## Discussion

We describe 3 cases of CD caused by ACTH-secreting pituitary macroadenomas, all displaying overt clinical features of hypercortisolism and associated complications including hypertension, diabetes mellitus, and reduced bone density [2].

According to standard diagnostic guidelines, clinical suspicion was followed by confirmatory biochemical tests and imaging [10]. Biochemical assessment included UFC, midnight serum cortisol, ACTH levels, DST, and assessment of pituitary function [10]. MRI played an essential role in tumor localization, assessment of invasion, and surgical planning [2].

Hwang et al and Selvais et al [5, 6] found higher ACTH levels in macroadenomas than microadenomas, while cortisol levels were comparable between the two groups. Our cases demonstrated both higher ACTH and cortisol levels with larger tumor dimensions, consistent with other studies [7, 8].

Our findings are in contrast with some reports suggesting that patients with macroadenomas may present with milder disease manifestations than patients with microadenomas [4]. Although similar common clinical features were observed, complications such as hypertension were found in less than 50% of patients [4]. Other studies have shown a higher prevalence of visual field deficits with macroadenomas, which our study supports [6]. This variability across different studies may be attributed to referral bias, where tertiary centers encounter patients at various stages of disease, and heterogeneity in tumor functionality, where macroadenomas do not necessarily produce more cortisol resulting in milder clinical disease [4].

MRI demonstrated macroadenomas (≥10 mm) [3] with cavernous and/or sphenoid sinus invasion in all cases. Mass effect was observed in one patient resulting in cranial nerve III palsy, while the others had visual field deficits secondary to optic chiasm compression [11]. Hypopituitarism was found in all patients, likely due to both pituitary gland compression and suppressive effects of hypercortisolism [11]. Mild hyperprolactinemia in all cases was consistent with pituitary stalk effect [11].

Neuropsychiatric deficits, including depression, emotional lability, anxiety, psychosis, hippocampal atrophy, and neurocognitive deficits, are known complications of hypercortisolism [1, 2]. Two patients were affected, with one experiencing severe depression and another with generalized cerebral atrophy on MRI [2].

All patients underwent surgical resection, the first-line treatment for functional adenomas [4]. One patient achieved remission, while the other 2 developed recurrent hypercortisolism, due to tumor regrowth, after initial biochemical normalization. Published data indicate lower remission rates and higher recurrence rates with macroadenomas compared to microadenomas, emphasizing the importance of long-term biochemical and radiological follow-up [9, 12]. One patient developed acute cortisol withdrawal syndrome postoperatively, which underscores the importance of individualized cortisol replacement therapy [13].

Medical therapy may be used to reduce cortisol levels as primary treatment or adjunctive treatment postoperatively. In our setting, this therapy is limited to cabergoline, ketoconazole, and mifepristone. Additional medical therapies including pasireotide are not widely available in South Africa's public health care system [12]. One patient received cabergoline and ketoconazole for 6 months prior to surgery, while the others underwent primary TSS.

Macroadenomas are associated with higher Ki-67 proliferation indices (>5%) and higher recurrence rates, suggesting its potential as a prognostic marker [14]. However, 2 of our patients had low indices (≤2%) yet recurrence still occurred in 1 patient. This suggests that Ki-67 index alone is not adequate to predict outcomes.

Limitations of this case series include the small sample size and limited long-term data for 1 patient; however, these cases still highlight the significant clinical presentations and their management.

ACTH-secreting pituitary macroadenomas are an uncommon cause of CD. This series expands the clinical spectrum by demonstrating that patients may present with overt disease and severe complications. We emphasize the importance of early recognition, first-line surgical management, and long-term follow-up. To our knowledge, this is the first reported case series from Sub-Saharan Africa, offering regional insights that contribute to global knowledge of this condition.

## Learning points

- ACTH-secreting pituitary macroadenomas are uncommon but can present with severe clinical features of CD.
- Macroadenomas often have complications of a mass effect and hypopituitarism.
- Surgical resection remains first-line treatment, but recurrence rates are higher in macroadenomas, emphasizing the need for lifelong follow-up.
- Corticosteroid replacement must be individualized.
- A Ki-67 proliferation index is not a reliable prognostic tool, as recurrence can still occur with a low index. Postoperative imaging and the presence of residual tumor should be considered in prognostic assessment.

## Contributors

All authors made individual contributions to authorship. C.I. and N.H. contributed to data collection, interpretation of endocrine investigations, and preparation of case descriptions. M.C.S. was involved in clinical evaluation, endocrine management, and directly managed the patients during their inpatient admission. D.R. was responsible for the neurosurgical management, including performing surgical procedures. R.D. contributed to radiological interpretation and imaging analysis. D.Z. contributed to histopathological analysis. All authors reviewed and approved the final manuscript.

## Data Availability

Some or all datasets generated during and/or analyzed during the current study are not publicly available but are available from the corresponding author on reasonable request.

## Abbreviations

ACTH: adrenocorticotropin
CCF: congestive cardiac failure
CD: Cushing disease
CS: Cushing syndrome
DST: dexamethasone suppression test
DXA: dual-energy x-ray absorptiometry
FSH: follicle-stimulating hormone
FT4: free thyroxine
HbA_1c_: glycated hemoglobin A_1c_
ICA: internal carotid artery
MRI: magnetic resonance imaging
LH: luteinizing hormone
RR: reference range
TSH: thyrotropin
TSS: transsphenoidal surgery
UFC: urinary free cortisol
